# How to Utilise Fat and Fish-Bones?

**Published:** 1915-04-17

**Authors:** 


### THE EDITOR'S LETTER-BOX.

### How to Utilise Fat and Fish-Bones?

*To the Editor of* The Hospital.

Sir,—In the times of financial difficulty through which hospitals are passing it is more than ever necessary to avoid waste of any kind. I am informed that in large hotels and restaurants all waste fat is carefully collected, and either made into common soap or sold. I have also heard that fish-bones, where the consumption of fish is considerable, are well worth collecting, and that there is a ready market for them. But the difficulty is to obtain precise information as to the best practical means of dealing with these by-products.

Probably the knowledge is not possessed by a great number of people, and in our district I know of no one who can give the information. I therefore write in the hope that some of your readers may be able to assist by advice in a matter which is not only of great interest but of great importance to hospitals.—I am, yours truly,

An Infirmary President.

